# Sub-Toxic Exposure to DEPs and PM_2.5_ Impairs Dendritic Cell Function Through Intracellular Particle Accumulation

**DOI:** 10.3390/jox15050142

**Published:** 2025-09-08

**Authors:** Yuki Nakahira, Daisuke Otomo, Tomoaki Okuda, Akira Onodera

**Affiliations:** 1 Department of Pharmaceutical Sciences, Kobe Gakuin University, Kobe 650-8586, Japan; 2 Faculty of Science and Technology, Keio University, Yokohama 223-8522, Japan; okuda@applc.keio.ac.jp

**Keywords:** PM_2.5_, diesel exhaust particles, dendritic cells, TLR7 signalling, lysosomal dysfunction

## Abstract

Air pollution, particularly exposure to fine particulate matter (PM_2.5_), poses a substantial risk to human health. Diesel exhaust particles (DEPs), a major constituent of PM_2.5_, contain chemically reactive components that promote inflammation, oxidative stress, and immune dysfunction. Although the acute toxicity of PM_2.5_ and DEPs has been extensively studied, their effects under “sub-toxic” conditions—defined here as exposures that do not cause measurable cytotoxicity based on LDH release but still impair cellular function—remain poorly understood. This study investigated the impact of low-toxicity exposure to DEPs and PM_2.5_ on dendritic cell (DC) function using the human plasmacytoid DC-like cell line PMDC05. Cells exposed to DEPs or PM_2.5_ exhibited minimal cytotoxicity but accumulated intracellular particles, resulting in impaired endocytosis, phagocytosis, and interferon gene expression upon TLR7 stimulation. These functional impairments were not observed following TLR4 stimulation, suggesting a selective disruption of endolysosomal signalling. The findings demonstrate that DEPs and PM_2.5_ can impair innate immune responses without inducing cell death, likely through lysosomal overload and altered intracellular trafficking. This study identifies a non-cytotoxic pathway through which particulate air pollution may compromise antiviral immunity, thereby increasing susceptibility to infection in polluted environments. Strategies aimed at preserving lysosomal integrity and dendritic cell function may help mitigate the immunotoxic effects of airborne particles.

## 1. Introduction

Air pollution, particularly exposure to fine particulate matter (PM_2.5_), poses a serious global health threat. Diesel exhaust particles (DEPs), a major contributor to urban air pollution, are a key component of ambient PM_2.5_ in the environment. Standardised DEP samples used in toxicological studies, such as SRM 2975 and NIST DEP, have been shown to exhibit chemical compositions and biological activities similar to those of PM_2.5_ found in urban environments [1]. The harmful constituents of PM_2.5_, including organic compounds (e.g., PAHs and nitro-PAHs), metals, and carbonaceous particles, are derived from DEPs and are recognized as key drivers of inflammation, oxidative stress, and DNA damage [2]. Epidemiological studies have consistently shown that exposure to PM_2.5_ increases the risk of both respiratory and cardiovascular diseases. Short-term exposure (hours to days) is associated with exacerbation of airway inflammation and hyper-responsiveness, as well as more frequent asthma attacks. Long-term exposure (years) contributes to lung function decline and the development or progression of chronic obstructive pulmonary disease (COPD) [3]. A two-sample Mendelian randomisation analysis identified a significant causal relationship between PM_2.5_ exposure and asthma and COPD, among eight pulmonary diseases examined [4].

Regarding cardiovascular effects, short-term PM_2.5_ exposure elevates the incidence of myocardial infarction, angina, arrhythmia, and stroke [5]. Long-term exposure contributes to the progression of atherosclerosis and increases mortality from cardiovascular disease [5]. A 10 μg/m^3^ increase in PM_2.5_ concentration is associated with a 6–17% increase in all-cause mortality and a 1.1–1.2-fold higher risk of myocardial infarction and ischemic heart disease [5]. Recent evidence further indicates that specific components of PM_2.5_, such as elemental carbon and secondary inorganic aerosols, are particularly associated with increased myocardial infarction risk, and that genetic susceptibility may amplify these adverse effects [6]. These impacts are especially pronounced in individuals with pre-existing hypertension, diabetes, or cardiovascular conditions. Elevated PM_2.5_ levels are also linked to increased outpatient visits, emergency department usage, and hospitalisations due to acute respiratory infections, particularly among children and the elderly. For every 10 µg/m^3^ increase in PM_2.5_ exposure, the risk of infection with Pseudomonas aeruginosa and MRSA increases by 24–68% [7]. Recent evidence further shows that in pediatric populations, specific exposure windows characterised by interactions between elevated PM_2.5_ levels and certain meteorological conditions can trigger epidemics of Mycoplasma pneumoniae pneumonia, with age-stratified analyses identifying heightened susceptibility among younger children [8]. Animal studies have demonstrated that exposure to PM_2.5_ impairs macrophage function and reduces bacterial clearance, thereby increasing susceptibility to Listeria, Staphylococcus aureus, Klebsiella pneumoniae, and Streptococcus pneumoniae [9]. More recent work has further shown that PM_2.5_ exposure can promote susceptibility to Pseudomonas aeruginosa infection in mice by driving the expansion of PD-L1 (high) neutrophils, highlighting an additional immunosuppressive pathway through the neutrophil-mediated regulation of host defense [10]. These findings suggest that airborne particulate matter may directly disrupt immune cell function, leading to dysregulated immune responses and heightened vulnerability to infection.

Dendritic cells (DCs) serve as a vital link between innate and adaptive immunity. Surface expression of Toll-like receptors (TLRs 1/2/4/6) and endosomal localisation of TLRs (3/7/8/9), along with C-type lectin receptors such as Dectin-1, Dectin-2, and Mincle, enable DCs to effectively internalise tumor-associated antigens (peptides, RNA, and DNA). These processes activate downstream signalling pathways, including the MyD88/TRIF and Syk-PLCγ pathways, which in turn activate NF-κB and MAP kinases. Activated DCs upregulate MHC class I/II and costimulatory molecules (CD80/CD86), facilitating antigen presentation and subsequent T cell activation [11,12,13]. Exposure of human blood-derived CD1c^+^ DCs to urban particulate matter for six hours has been shown to induce IFN-γ, IL-13, and IL-17A production and promote differentiation of CD4^+^ memory T cells into Th1, Th2, and Th17 subsets in an MHC class II-dependent manner [14]. In line with these findings, transition metal-containing particulate matter has been reported to promote robust Th1 and Th17 inflammatory responses through monocyte activation, with the magnitude of the response depending on the presence of specific organic and inorganic compounds [15]. In contrast, pro-oxidative chemicals in DEPs induce oxidative stress in mouse bone marrow-derived DCs (BMDCs) and human DC lines, suppressing DC maturation and IL-12 production following lipopolysaccharide (LPS) stimulation [16]. Consistent with these immunomodulatory effects, DEP exposure has also been shown to facilitate the development of allergic asthma in response to otherwise sub-sensitizing doses of soybean allergens, suggesting that DEPs can lower the activation threshold for allergic sensitization and exacerbate airway inflammation [17]. These findings suggest that DEPs and PM_2.5_ have variable effects on DC function, depending on the exposure conditions and the degree of cytotoxicity. However, how DC function is altered under sub-toxic conditions—defined here as exposures that do not cause measurable cytotoxicity based on LDH release but still impair cellular function—involving intracellular particle accumulation remains poorly understood.

Particles internalised by cells are believed to localise to lysosomes, similar to other foreign materials. In mouse BMDCs, polystyrene particles with diameters of 20 and 1000 nm accumulate in lysosomes, with smaller particles accumulating more rapidly and abundantly [18]. Consistently, studies using gold nanoparticles have shown that surface modifications, such as monosaccharide coatings, can modulate their intracellular trafficking pathways within dendritic cells, influencing the extent and kinetics of lysosomal localisation [19]. Carbonaceous particles in PM_2.5_ and DEPs resist degradation even in acidic lysosomal environments and may persist intracellularly. Considering that DCs typically survive for 3–9 days, it is plausible that particle-laden DCs remain at the frontline of innate immune defense throughout this period [20]. Consistent with the notion of long-lasting intracellular persistence, alterations in autophagy have been observed in the lung tissues of mice even 30 days after co-exposure to carbon black and metal ions, suggesting sustained effects on lysosomal function and immune regulation [21]. Previous studies have shown that exposure to vehicle exhaust particulates reduces lysosomal acid stability in airway epithelial cells [22].

In this study, the human plasmacytoid DC-like cell line PMDC05 was used to investigate how the intracellular accumulation of subtoxic DEPs and PM_2.5_ affects fundamental DC functions, including endocytosis, phagocytosis, and antiviral interferon (IFN) responses. We hypothesised that PM_2.5_ and DEPs exert multi-phase biological effects: an early phase characterised by inflammation shortly after exposure, and a later phase driven by particle retention and accumulation. At the cellular level, we further propose that similar multi-phase effects occur, in which intracellular particle uptake and accumulation lead to the functional impairment of dendritic cells, even under sub-toxic conditions. For the first time, the findings demonstrate that intracellular particle accumulation impairs DC immune function under non-cytotoxic conditions.

## 2. Materials and Methods

### 2.1. Cell Culture

PMDC05 cells, a human plasmacytoid dendritic cell (pDC) line originally established from leukaemia blasts of an HLA-A*02:06/24:02 patient with pDC leukaemia, were generously provided by Dr. Takashi Ushiki of the Graduate School of Health Sciences, Niigata University. The establishment and characterisation of this cell line were previously described by Narita et al. [23], in which leukaemia cells were isolated from peripheral blood, cultured in RPMI-1640 medium supplemented with fetal bovine serum and cytokines, and maintained under conditions promoting pDC differentiation and proliferation. Cells were maintained in Iscove’s Modified Dulbecco’s Medium (IMDM; Invitrogen, Grand Island, NY, USA) supplemented with 10% heat-inactivated HyClone™ Bovine Growth Serum (BGS; Cytiva, Marlborough, MA, USA) and 1% antibiotic–antimycotic solution (Nacalai Tesque, Kyoto, Japan). The BGS was heat-inactivated at 56 °C for 30 min before use. For routine subculture, cells were seeded at a density of 1 × 10^6^ cells/mL and passaged when the cell density reached approximately 5 × 10^6^ cells/mL. Passaging was performed once or twice weekly, depending on cell growth. For particle exposure experiments, the BGS concentration was reduced to 0.5% to minimise the formation of a hard protein corona. In general, serum proteins, such as albumin and immunoglobulins, rapidly and stably bind to the surface of nanoparticles, forming a hard corona that significantly alters particle–cell interactions and cellular responses [24,25]. Thus, the BGS levels were minimised during exposure to better assess the intrinsic biological effects of the particles. All procedures were conducted in a humidified incubator maintained at 37 °C with 5% CO_2_.

### 2.2. Preparation of DEPs and PM_2.5_ Suspensions

DEPs (NIST SRM 2975), derived from industrial forklifts, were suspended in sterile phosphate-buffered saline (PBS; pH 7.4). The suspension was vortexed and sonicated to minimise particle aggregation before use. Detailed information on this DEP reference material is available from the National Institute of Standards and Technology (NIST) at https://www.nist.gov/srm (accessed on 1 September 2025), and the Certificate of Analysis can be found at https://tsapps.nist.gov/srmext/certificates/2975.pdf (accessed on 1 September 2025). Atmospheric PM_2.5_ samples (sample code: JP-PM_2.5_-23) were collected from ambient air above the rooftop of a building at Keio University (Yokohama, Japan) between 19 April and 18 June 2021, using a high-volume cyclone sampler (K-RiC), a device originally developed for particulate matter collection [26]. This cyclone-based method yields PM_2.5_ in powder form, thereby reducing sampling artefacts commonly associated with filter-based methods. The collected PM_2.5_ was suspended in PBS, vortexed, and sonicated using the same protocol as that for DEP. Working suspensions were freshly prepared in culture medium containing 0.5% BGS for both DEPs and PM_2.5_ and used immediately in cell exposure experiments.

### 2.3. Lactate Dehydrogenase (LDH) Cytotoxicity Assay

PMDC05 cells were seeded at a density of 1 × 10^6^ cells/well in 24-well plates (BM Equipment Co., Ltd., Tokyo, Japan) and incubated for 18 h. The cells were then exposed to either 50 µg/mL of DEPs or 100 µg/mL of PM_2.5_ for 24 h. The dispersion medium for both particle types, PBS, was used as the control. For each experimental condition, cells were cultured in three wells, and the supernatants from each well were collected and analysed separately in triplicate. Following incubation, the culture supernatants were centrifuged at 800× *g* for 5 min at 4 °C using a refrigerated centrifuge (Beckman Coulter, Brea, CA, USA). The resulting supernatants were used to assess LDH activity. For total LDH determination, unexposed cells were lysed with digitonin (100 µg/mL) for 10 min at 37 °C and then subjected to the same centrifugation protocol. LDH activity was measured using the LDH-Cytotoxic Test Wako kit (Fujifilm Wako Pure Chemical Co., Osaka, Japan) according to the manufacturer’s instructions. Absorbance was measured at 560 nm with a reference wavelength of 620 nm using a multimode microplate reader (Synergy LX, Agilent Technologies, Santa Clara, CA, USA). Assays were conducted in 96-well plates, with each sample measured in technical triplicate. The entire experiment was repeated three times (*n* = 3). Statistical analyses were performed using the Mann–Whitney U test for pairwise comparisons as a nonparametric method, with Bonferroni correction for multiple comparisons, implemented in EZR software (version 1.68) [27]. Statistically significant differences are indicated in the figure legends.

### 2.4. Assessment of DEPs and PM_2.5_ Uptake by Flow Cytometry and Morphological Observation

PMDC05 cells were seeded at a density of 1 × 10^6^ cells/mL into 35 mm glass-bottom dishes (ibidi GmbH, Gräfelfing, Germany) and incubated for 18 h. Cells were then exposed to DEP, PM_2.5_, or PBS, serving as the vehicle control. After 24 h of exposure, bright-field images were captured using a digital single-lens reflex camera (Canon Inc., Tokyo, Japan) under identical exposure settings. Immediately following imaging, cells, including any sedimented particles, were harvested by centrifugation at 800× *g* for 8 min. The resulting cell pellet was resuspended in PBS containing 1% bovine serum albumin and 2 mM EDTA and then passed through a 70 µm nylon mesh filter (Falcon™, Corning Inc., Corning, NY, USA). Flow cytometric analysis was performed using a FACSCanto system (BD Biosciences, San Jose, CA, USA), and the data were analysed using the Floreada.io Analysis Suite (Floreada.io; https://floreada.io/analysis (accessed on 2 September 2025)). Cells were gated based on forward scatter (FSC) and side scatter (SSC), and granularity was assessed via SSC distribution. Histogram plots were normalised to “Percent Max” on the y-axis, with SSC values plotted along the x-axis. Statistical comparisons were performed using the Wilcoxon signed-rank test with continuity correction for paired data. All statistical analyses were conducted using the EZR software. Statistically significant differences were directly annotated on the graphs. The entire experiment was independently repeated three times for each particle type.

### 2.5. Assessment of Phagocytic Activity Following DEP or PM_2.5_ Exposure Using Fluorescent Particles

PMDC05 cells were seeded at a density of 1 × 10^6^ cells/well into 12-well plates (BM Equipment Co., Ltd., Tokyo, Japan) and incubated for 18 h. Cells were then exposed to DEPs (5 or 50 µg/mL), PM_2.5_ (100 or 150 µg/mL), or PBS for 48 h. Following this pre-exposure, cells were incubated for 24 h with one of the following fluorescent particles: Sicastar-redF, COOH, 300 nm (Micromod Partikeltechnologie GmbH, Rostock, Germany) at a concentration of 100 µg/mL (detected via the PE channel), or Fluoresbrite YG Carboxylate Microspheres, 0.75 µm (Polysciences Inc., Warrington, PA, USA) at a concentration of 1 × 10^8^ particles/mL (detected via the FITC channel).

Prior to administration, both types of particles were opsonised by incubating them with fetal calf serum at 37 °C for 30 min to enhance cellular uptake. As a positive control for uptake inhibition, Cytochalasin D (Cyto D; FUJIFILM Wako Pure Chemical Corporation, Osaka, Japan) was added at a final concentration of 1 µM to inhibit actin polymerisation. After particle exposure, cells were harvested and stained with 7-Aminoactinomycin D (7-AAD, Thermo Fisher Scientific, Waltham, MA, USA) according to the manufacturer’s protocol. Flow cytometric analysis was performed using the same instrument settings described in Section 2.4. Briefly, viable cell populations were first gated based on FSC and SSC, followed by FSC vs. 7-AAD gating to select live cells. Histograms of particle uptake were generated using the PE channel (for 300 nm silica particles) or FITC (for 750 nm polystyrene particles) within the Floreada.io Analysis Suite. Statistical comparisons were conducted using the Wilcoxon signed-rank test for paired data, implemented in EZR software. Statistically significant differences were directly annotated on the graphs.

### 2.6. Quantitative RT-PCR Analysis of Interferon Gene Expression Following Toll-like Receptor (TLR) Stimulation

PMDC05 cells were seeded at a density of 1 × 10^6^ cells/mL into 60 mm culture dishes (AGS Techno Glass, Shizuoka, Japan) and incubated for 18 h. Cells were then exposed to 50 µg/mL of DEP, 100 µg/mL of PM_2.5_, or PBS. After 48 h, the cells were stimulated with either 4 ng/mL of Lipid A (Peptide Institute Inc., Osaka, Japan), a TLR4 ligand; 5 µg/mL of Gardiquimod (GDQ; Enzo Life Sciences, Inc., Farmingdale, NY, USA), a TLR7 agonist; or treated with DMSO as a vehicle control. After 6 h of stimulation, total RNA was extracted using TRI Reagent (Molecular Research Center, Cincinnati, OH, USA) according to the manufacturer’s protocol. RNA concentrations were measured using a NanoDrop 1000 Spectrophotometer (Thermo Fisher Scientific, Waltham, MA, USA). Genomic DNA was removed, and complementary DNA (cDNA) was synthesised using the ReverTra Ace^®^ qPCR RT Master Mix with gDNA Remover (Toyobo Co., Ltd., Osaka, Japan) according to the manufacturer’s instructions. Quantitative PCR (qPCR) was conducted using the THUNDERBIRD SYBR qPCR Mix (Toyobo Co., Ltd., Osaka, Japan) on an ABI 7500 Fast Real-Time PCR System (Applied Biosystems, Foster City, CA, USA) to quantify mRNA expression levels of IFN-β, IFN-α1/13, and IFN-α2. Primer sequences were designed using OriGene Technologies (Rockville, MD, USA; www.origene.com). Glyceraldehyde-3-phosphate dehydrogenase (GAPDH) was used as the internal reference gene. Data were analysed using the ΔΔCt method. Each qPCR sample was run in triplicate wells. The entire experiment was independently repeated three to five times. Statistical analysis was performed using the Wilcoxon rank sum test with Easy R software. Statistically significant differences are indicated in the figure legends.

## 3. Results

### 3.1. Short-Term Exposure to DEPs and PM_2.5_ Only Induces Subtoxic Cytotoxicity in PMDC05 Cells

To evaluate the potential cytotoxic effects of particle exposure, LDH release was quantified in PMDC05 cells following 24 h of treatment with either DEPs (50 µg/mL) or PM_2.5_ (100 µg/mL). Both DEPs and PM_2.5_ are dark-coloured particulate suspensions at high concentrations that can interfere with absorbance-based assays. Under the conditions used in this study, background interference was minimised, enabling reliable LDH measurement.

**Figure 1:**
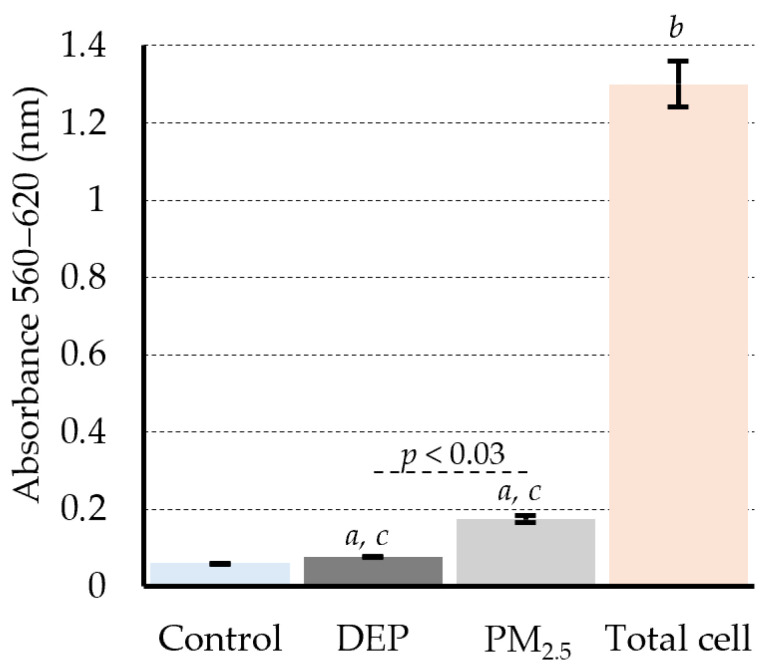
Low levels of LDH release from PMDC05 cells exposed to DEPs or PM_2.5_. PMDC05 cells were exposed to 50 µg/mL of DEPs or 100 µg/mL of PM_2.5_ for 24 h. The LDH activity in the culture supernatant was measured using the LDH-Cytotoxic Test Wako kit. The total LDH release was determined in digitonin-treated cells. Bars represent the mean ± standard deviation (SD) from three independent experiments. Each condition was assayed in triplicate. *a p* < 0.03 vs. control; *b p* < 0.01 vs. control; *c p* < 0.01 vs. total cell.

Statistical analysis revealed a slight but significant increase in LDH activity in the PM_2.5_-exposed group compared with the PBS control group (Figure 1). However, the overall cytotoxicity level remained low across all groups. LDH release, normalised to total cellular LDH, remained below 7% in DEP-exposed cells and 14% in PM_2.5_-exposed cells, compared with 4.6% in the control group. These results indicate that under the tested conditions, both particle types exert only minimal cytotoxic stress on PMDC05 cells, allowing subsequent functional assays to be conducted without interference from excessive cell death.

### 3.2. Dendritic Cell Model PMDC05 Visibly Uptakes DEPs and PM_2.5_ Particles Without Signs of Cytotoxicity

**Figure 2:**
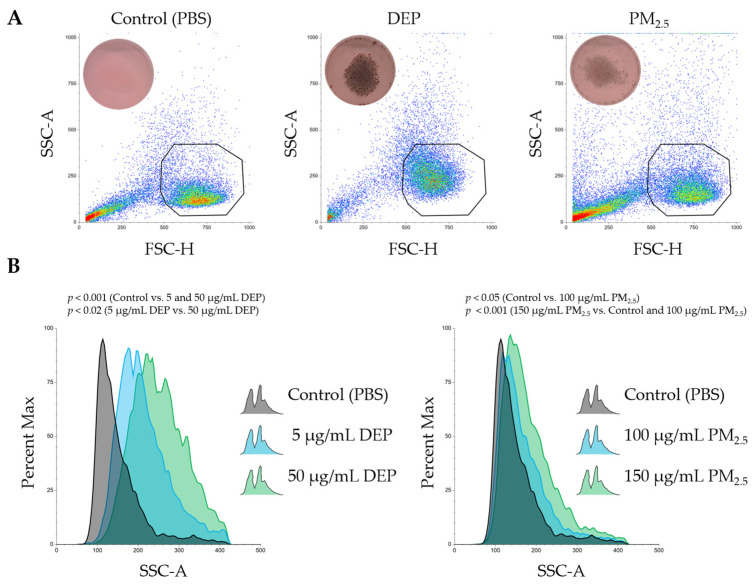
PMDC05 cells internalise particulate matter from DEPs and PM_2.5_, resulting in increased internal complexity, as reflected by SSC profiles. (**A**) Representative dot plots showing forward scatter height (FSC-H) versus side scatter area (SSC-A) of PMDC05 cells treated with PBS (Control), DEP, or PM_2.5_ for 24 h. The upper left of each plot shows bright-field images captured with a digital single-lens reflex camera. Intracellular black particles are visible in cells exposed to DEPs and PM_2.5_. The gated regions indicate the main cell populations analysed. (**B**) SSC-A histograms of PMDC05 cells exposed to DEPs (left) and PM_2.5_ (right). The Y-axis represents normalised cell counts (Percent Max), and the X-axis shows SSC-A values reflecting cellular granularity. Particle uptake is associated with a pronounced rightward shift in the SSC-A distribution. DEP exposure concentrations were 5 µg/mL and 50 µg/mL, and PM_2.5_ exposure concentrations were 100 µg/mL and 150 µg/mL. Statistically significant differences were directly annotated on the graphs.

PMDC05 cells, a well-established model of human plasmacytoid dendritic cells (pDCs), were used to evaluate particulate uptake by dendritic cells. After 24 h of exposure to either DEPs or PM_2.5_, dark intracellular inclusions, presumed to be internalised particles, were observed through digital photography (Figure 2A). This visual evidence indicated effective uptake of both particle types, as exposed cells appeared darker than those in the PBS control group. Despite this evident internalisation, no morphological features indicative of necrosis or cell swelling were observed, either visually or by flow cytometric analysis (Figure 2A). Consistent with these observations, LDH levels in culture supernatants remained unchanged compared with the control group, indicating no significant membrane damage or cytotoxicity under the experimental conditions (Figure 1).

To quantify changes in cellular granularity and internal complexity associated with particle uptake, SSC-A (Side Scatter Area) profiles were analysed by flow cytometry. Exposure to both DEPs and PM_2.5_ induced a marked rightward shift in SSC-A histograms, reflecting increased intracellular complexity (Figure 2B). These changes were concentration-dependent and reproducible across independent experiments.

Taken together, these findings demonstrate that PMDC05 cells efficiently phagocytose both DEPs and PM_2.5_ particles without triggering detectable cytotoxic responses.

### 3.3. Exposure to DEPs and PM_2.5_ Suppresses Both Endocytosis and Phagocytosis in PMDC05 Cells

**Figure 3:**
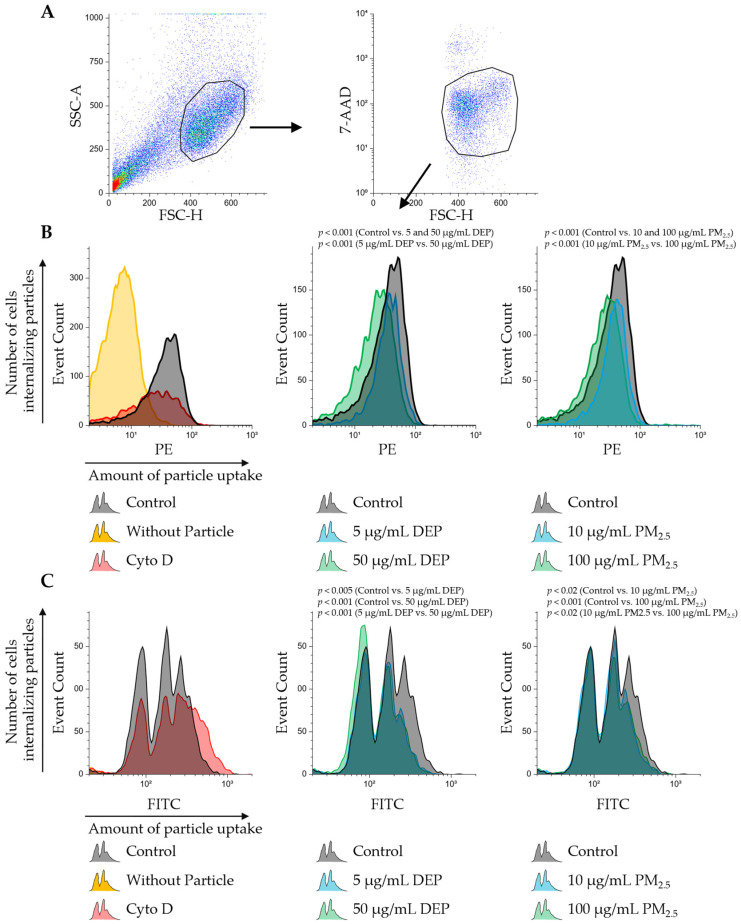
Uptake of 300 nm and 750 nm fluorescent particles is suppressed by DEP and PM_2.5_ pre-exposure in PMDC05 cells. (**A**) Representative gating strategy used for flow cytometric analysis. PMDC05 cells were first gated based on forward scatter height (FSC-H) versus side scatter area (SSC-A), followed by selection of live cells using FSC-H versus 7-aminoactinomycin D (7-AAD) staining. (**B**) Histogram plots showing uptake of 300 nm fluorescent silica particles (Sicastar-redF; detected in the phycoerythrin [PE] channel) by PMDC05 cells. Cells were pre-exposed for 48 h to PBS (control), DEPs (5 or 50 µg/mL), or PM_2.5_ (10 or 100 µg/mL), then incubated with opsonised particles for 24 h. Particle uptake was markedly reduced in cells pre-exposed to DEPs or PM_2.5_. A Cytochalasin D (CytD)-treated group was included as a positive control for inhibition of uptake. The PE fluorescence intensity reflects the extent of particle internalisation. (**C**) Histogram plots showing the uptake of 750 nm fluorescent polystyrene particles (Fluoresbrite YG; detected in the fluorescein isothiocyanate [FITC] channel) under the same pretreatment and exposure conditions as in (**B**). Similar inhibition of phagocytosis was observed following exposure to DEPs and PM_2.5_.

To assess the effect of pre-exposure to DEPs or PM_2.5_ on particle uptake, PMDC05 cells were incubated with fluorescent silica particles (300 nm) or polystyrene particles (750 nm) 48 h after pretreatment. Dead cells were excluded using 7-AAD staining, and only viable cell populations were analysed (Figure 3, Supplementary Figure S1). Particle uptake is size-dependent: particles smaller than 0.5 µm typically enter cells via clathrin- or caveolin-mediated endocytosis, whereas larger particles greater than 0.5 µm are internalised through actin-dependent phagocytosis [28]. To distinguish between these pathways, particles of 300 nm (endocytosis-prone) and 750 nm (phagocytosis-prone) were used. PMDC05 cells efficiently internalised 300 nm particles, as evidenced by a rightward shift in fluorescence intensity relative to the no-particle control (Figure 3B). Treatment with cytochalasin D substantially reduced the number of particle-positive cells, validating the role of actin in particle uptake. Pre-exposure to either DEPs or PM_2.5_ significantly reduced the number of particle-positive cells, with greater suppression observed at the higher concentration, indicating reduced endocytic activity following pretreatment (Figure 3B).

In contrast, the uptake of 750 nm particles produced histogram peaks (Figure 3C), likely corresponding to low-, mid, and high-uptake cell populations. Cytochalasin D treatment nearly abolished particle internalisation. Pre-exposure to DEPs or PM_2.5_ significantly reduced the proportion of cells with mid- and high-particle loads. DEP exposure nearly eliminated the high-uptake population, whereas PM_2.5_ primarily suppressed the high-load peak. These results suggest that the phagocytic capacity is differentially suppressed depending on the particle type and treatment condition (Figure 3C).

Although the histograms of the 300 nm particle uptake did not resolve into distinct peaks—likely due to limitations in fluorescence intensity or detector resolution—the overall trend of reduced uptake was evident (Figure 3B,C). Importantly, after the second exposure, no significant cell death was observed for either particle type (Supplementary Figure S1), confirming that the suppression of uptake was not a consequence of cytotoxic effects.

### 3.4. DEPs and PM_2.5_ Selectively Inhibit Interferon Gene Expression Induced by Virus-like Stimulation Through TLR7

**Figure 4:**
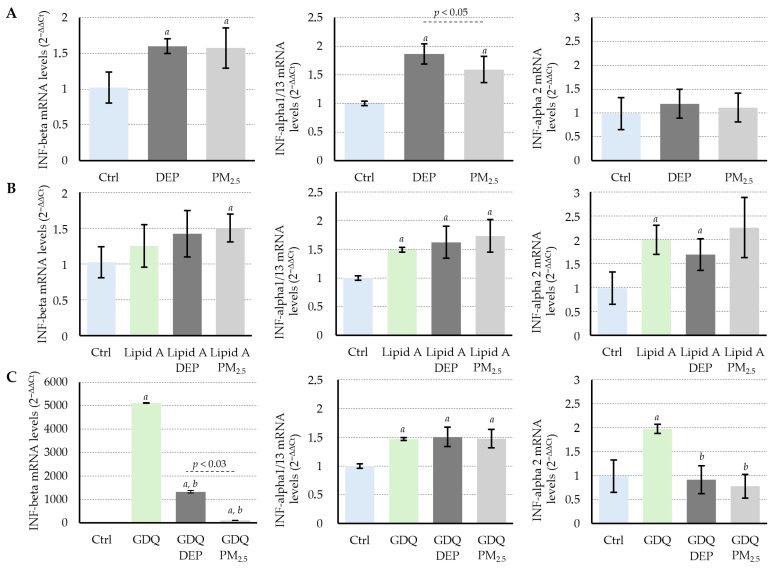
Pretreatment with DEPs and PM_2.5_ suppresses GDQ-induced interferon mRNA expression in PMDC05 cells. (**A**) Baseline mRNA expression levels of IFN-β, IFN-α1/13, and IFN-α2 following 52 h exposure (48 h + 6 h) to DEPs (50 µg/mL), PM_2.5_ (100 µg/mL), or PBS in PMDC05 cells. (**B**) mRNA expression of IFN genes after TLR4 stimulation with Lipid A (4 ng/mL) and 48 h pretreatment with DEP, PM_2.5_, or PBS. (**C**) mRNA expression following TLR7 stimulation with Gardiquimod (GDQ; 5 µg/mL) and 48 h pretreatment with DEP, PM_2.5_, or PBS. Gene expression levels were normalised to GAPDH and calculated using the ΔΔCt method. Bars represent mean ± SD from three to five independent experiments. Each condition was assayed in triplicate. *a p* < 0.001 vs. control; *b p* < 0.001 vs. GDQ.

This study evaluated the effects of DEPs and PM_2.5_ on the induction of interferon gene expression triggered by TLR ligand stimulation. First, PMDC05 cells were exposed to DEPs or PM_2.5_ alone for a total of 52 h, consisting of 48 h of initial exposure followed by an additional 6 h without any stimulation. This treatment significantly increased the mRNA expression of IFN-β and IFN-α1/13 (Figure 4A). In a separate experiment, PMDC05 cells were pre-exposed to DEPs or PM_2.5_ for 48 h and then stimulated for 6 h with Lipid A, a cell membrane-localised TLR4 ligand. Lipid A alone significantly increased the expression of IFN-α1/13 and IFN-α2 (Figure 4B). Pretreatment with DEPs or PM_2.5_ did not significantly alter this induction, although a trend towards increased expression of IFN-β and IFN-α1/13 with DEPs and of IFN-β, IFN-α1/13, and IFN-α2 with PM_2.5_ was observed (Figure 4B). In contrast, when cells pre-exposed to DEPs or PM_2.5_ were stimulated with GDQ, an endosome/lysosome-localised TLR7 ligand, GDQ alone induced an approximately 5000-fold increase in IFN-β mRNA expression and a 1.5- to 2-fold increase in IFN-α1/13 and IFN-α2 expression (Figure 4C). However, in cells pre-exposed to DEPs or PM_2.5_ for 48 h, GDQ-induced mRNA expression of IFN-β and IFN-α2 was significantly suppressed, whereas no suppressive effect was observed for IFN-α1/13 (Figure 4C).

## 4. Discussion

This study demonstrated that sub-toxic exposure, defined here as DEP and PM_2.5_ concentrations that did not induce significant cytotoxicity relative to the PBS control, results in intracellular particle accumulation in human dendritic cell-like PMDC05 cells (Figure 1 and Figure 2). Despite its minimal cytotoxicity, TLR7 stimulation led to suppressed endocytic and phagocytic activity, along with significant inhibition of interferon gene expression (Figure 3, Figure 4 and Figure S1). These findings suggest that particulate matter may impair innate immune function without causing overt cytotoxic effects.

Particulate matter, such as DEPs and PM_2.5_, likely affects human health in at least two distinct phases: an early phase, occurring shortly after exposure, marked by cytotoxic and inflammatory injury, and a delayed phase, arising from particle accumulation, leading to immune dysfunction. As discussed in the Introduction, the latter phase has been associated with increased mortality from respiratory and cardiovascular diseases and heightened susceptibility to viral infections. DCs, which serve as central coordinators of innate and adaptive immunity, have a typical lifespan of approximately 3–9 days in vivo before undergoing apoptosis or being replaced by new cells derived from bone marrow precursors [20]. A previous study examining DC kinetics in murine lymphoid organs demonstrated that tissue-resident DCs in secondary lymphoid organs such as lymph nodes and spleen are maintained through continuous replenishment from circulating bone marrow-derived precursors, achieved via a tightly regulated balance of maturation, migration, and apoptosis [29]. A dendritic cell-like model was employed to investigate the effects of excessive intracellular particle accumulation on immune function in the absence of significant cytotoxicity. Although LDH assays confirmed minimal cell death following DEP and PM_2.5_ exposure, an apparent reduction in endocytic and phagocytic activity was observed, along with impaired TLR7-dependent interferon responses (Figure 1, Figure 3, Figure 4 and Figure S1). The fact that particle uptake was reduced but not completely abolished further supports the interpretation that this suppression results from functional impairment rather than overt cytotoxicity (Figure 3B,C). These results suggest that functional impairment was not the result of acute toxicity but rather of intracellular stress and homeostatic disruption induced by the sustained presence of internalized particles throughout the 24–48 h exposure period in our in vitro system. The findings provide experimental evidence that DCs, while physically intact, may become functionally compromised when overloaded with non-degradable particulate matter. This underscores the importance of considering the non-cytotoxic immunomodulatory effects of airborne particles in immunotoxicology and environmental health risk assessments.

In animal models, inhalation exposure to PM_2.5_ significantly impaired the phagocytic capacity of alveolar macrophages upon subsequent infection with Pseudomonas aeruginosa, as assessed by the Neutral Red Phagocytosis assay. Exposure to PM_2.5_ or P. aeruginosa alone triggered only mild inflammation, but their combination led to excessive activation of the mTOR signalling pathway, which in turn suppressed the expression of phagocytosis-related molecules such as LC3B, a microtubule-associated protein 1 light chain 3 beta and a marker of autophagosome formation [30]. Similarly, in a separate study, mice exposed to PM_2.5_ and subsequently infected with the H1N1 influenza virus exhibited delayed viral clearance, accompanied by reduced activation of the NLRP3 inflammasome and lower IL-1β production, indicating suppressed antiviral immune responses [31]. These studies strongly support the hypothesis that the intracellular accumulation of particulate matter impairs immune function independently of overt cytotoxicity, which aligns with the suppressed dendritic cell responses observed in the current study.

In another study using DEP, human DCs differentiated from CD34^+^ hematopoietic progenitor cells exhibited increased FITC–dextran uptake [32]. However, in that experiment, DEPs were immobilised on a Teflon filter, which prevented direct particle internalisation [32]. Therefore, the observed increase in uptake likely reflects an indirect enhancement of endocytic activity in response to DEPs, rather than actual particle uptake

Although DEPs and PM_2.5_ are both commonly categorised as combustion-derived particles, the present study revealed differences in their effects on dendritic cell function and fluorescence particle uptake. These differences cannot be fully explained by particle concentration alone. One approach to improving comparability is to align experimental exposure levels with environmental measurements. Nevertheless, both DEPs and PM_2.5_ are black particles that may interfere with colourimetric and fluorescence-based assays. To ensure data reliability, confirmation was first obtained that the particles did not produce false-positive signals in cell-free systems.

The differential effects of DEPs and PM_2.5_ on dendritic cell function are likely attributable to variations in their physicochemical properties, including particle size, surface charge, and chemical composition (Table 1 and Table S1). Although both particle types affect lysosomal function and interferon responses, they appear to do so via distinct mechanisms. Table 1 shows the chemical components known to affect lysosomal integrity, dendritic cell function, and immune modulation, with values normalised to the concentrations used in cellular assays. For DEP, data on total organic carbon, elemental carbon, Fe, and Zn were obtained from the literature, based on filter-collected particles that incorporated both soluble and insoluble fractions [1]. Endotoxin concentrations for DEPs were similarly cited from aqueous suspension measurements, which were reported near the detection limit [33]. The chemical composition of PM_2.5_ was based on direct measurements conducted at Keio University (Supplementary Table S1).

The component concentrations of DEPs were estimated based on representative values compiled from the published literature, whereas those of PM_2.5_ were directly determined by chemical analysis. See Supplementary Table S1 for full compositional data and PM_2.5_ analytical details.

Polycyclic aromatic hydrocarbons (PAHs) and quinones, commonly found in both DEPs and PM_2.5_, are known to impair mitochondrial and lysosomal integrity by generating reactive oxygen species (ROS). For example, exposure of RAW264.7 macrophage-like cells to aromatic and polar organic compounds extracted from DEPs resulted in increased superoxide production, decreased mitochondrial membrane potential, and reduced mitochondrial mass. Additionally, the opening of the mitochondrial permeability transition pore, which is sensitive to cyclosporin A, was induced, leading to mitochondrial swelling and depolarisation [34]. In a separate study using heated PM_2.5_ (h-PM_2.5_), in which volatile organic compounds were removed by thermal treatment, co-treatment with h-PM_2.5_ and quinones enhanced cell death and ROS production in human airway epithelial cells compared with PM_2.5_ alone [35]. These findings suggest that PAHs and quinones in DEPs and PM_2.5_ may induce subtoxic levels of ROS, leading to mitochondrial dysfunction and destabilisation of lysosomal membranes.

Black carbon, a major component of both DEPs and PM_2.5_, can be repeatedly internalised and accumulate in lysosomes, resulting in lysosomal overload. Although the detailed mechanisms remain unclear, previous findings reported that DEP exposure in airway epithelial cells leads to lysosomal accumulation and destabilisation of lysosomal pH [22]. During the degradation of iron-containing macromolecules, such as ferritin, lysosomes tend to accumulate redox-active labile iron, making them susceptible to oxidative stress. For instance, excessive ethanol intake increases hepatic iron accumulation in the lysosomal labile iron pool, leading to oxidative damage and apoptosis in hepatocytes. Quercetin has been shown to reduce lysosomal iron levels and suppress lysosomal membrane permeability (LMP) [36].

In alveolar macrophages from mice, nanoparticles such as ZnO, TiO_2_, and CeO_2_ have been reported to alter lysosomal ion permeability—particularly for potassium—leading to K^+^ influx into lysosomes, membrane hyperpolarisation, and induction of LMP [37]. Similar changes in LMP have been reported for other metal ions. For instance, oxidative stress-induced LMP is a key trigger for neuronal cell death. Accumulation of labile zinc in lysosomes has been observed following H_2_O_2_ exposure, and zinc exposure alone was sufficient to induce LMP. These effects have also been confirmed in isolated lysosomes [38]. To provide a mechanistic context, previous studies have identified multiple chemical and physical components of DEPs and PM_2.5_, including PAHs, quinones, black carbon, and metal-based nanoparticles, that can impair lysosomal and mitochondrial function via oxidative stress, ion imbalance, and persistent particle retention. Summarising these findings supports the biological plausibility of our observed lysosomal destabilisation in PMDC05 cells. Collectively, these findings indicate that PM_2.5_ may compromise lysosomal integrity through multiple pathways, involving both chemical and physical stressors.

DCs mature by internalising antigens and presenting them via MHC class II molecules, while gradually reducing their antigen uptake capacity during maturation [39]. Therefore, when interpreting reductions in phagocytosis and endocytosis, DC maturation, in addition to particle composition, should be taken into consideration. Even at low concentrations, LPS can provoke strong immune responses. For instance, primary human monocytes have been shown to produce TNF-α at a concentration of 0.0025 ng/mL and IL-6 at 0.005 ng/mL after exposure [40]. Therefore, prolonged exposure (48 h) to endotoxin-containing DEPs and PM_2.5_ may partially promote DC maturation. However, because phagocytic capacity was not entirely lost in the present study, the contribution of endotoxins is considered limited. Taken together, physical particle accumulation and lysosomal overload appear to be the primary drivers of the observed functional impairment.

The suppression of TLR7-dependent interferon responses observed after DEP and PM_2.5_ exposure is likely associated with the endosomal and lysosomal localisation of TLR7. These organelles serve as essential platforms for TLR7 signalling, and physical disruption of the lysosomal environment due to particle accumulation may directly impair receptor activation and downstream signalling [41,42]. TLR7 is synthesised as an inactive full-length protein and is subsequently cleaved by acidic proteases within endolysosomal compartments to generate an active C-terminal fragment, which binds its ligand and activates the MyD88–IRF7/NF-κB pathway [43]. This cleavage is pH-dependent, and alterations in lysosomal acidity significantly affect TLR7 activation. Indeed, the elevation of endolysosomal pH (deacidification) by human alpha-1 antitrypsin reduces the generation of cleaved TLR7, leading to decreased IL-12 and type I interferon production [44], providing direct evidence that lysosomal acidity is critical for TLR7 activation. This supports our hypothesis that particle-induced disruption of lysosomal pH could impair TLR7 signalling. In addition, studies using carbon nanotubes have demonstrated that unmodified multi-walled structures are highly resistant to enzymatic degradation and persist within cells for extended periods. Structural modifications, such as carboxylation or engineering of biconcave (red blood cell-like) shapes, improve their degradability and biocompatibility [45,46]. By analogy, the carbonaceous particles in DEPs and PM_2.5_ are likely to exhibit similar resistance to enzymatic degradation. The prolonged retention of these particles within lysosomes could disrupt lysosomal function and endosomal trafficking, thereby impairing lysosomal homeostasis and suppressing TLR7-mediated antiviral responses.

## 5. Conclusions

This study demonstrated that short-term, subtoxic exposure to DEPs and PM_2.5_ impairs the immune function of human dendritic cell-like PMDC05 cells without inducing overt cell death. Notably, the marked suppression of TLR7-mediated interferon responses resulting from intracellular particle accumulation reveals a non-cytotoxic immunosuppression mechanism triggered by ambient particulate matter. Given the central role of dendritic cells in orchestrating antiviral immunity and inflammatory responses, these findings suggest an under-recognised risk: that inhaled particles may selectively impair immune function even in the absence of cytotoxicity. This mechanism may contribute to the elevated incidence of respiratory infections in polluted environments.

Future strategies aimed at preserving dendritic cell function and maintaining lysosomal homeostasis may offer promising approaches for mitigating the immunotoxic effects of air pollution. Continued research into the long-term consequences of particle accumulation and the development of preventive interventions targeting dendritic cells could provide novel avenues for protecting the immune system from environmental dysfunction.

## Figures and Tables

**Table 1:** The major chemical components of DEPs and PM_2.5_ at treatment concentrations used in cellular assays (50 µg/mL for DEPs and 100 µg/mL for PM_2.5_).

Component	DEP	PM_2.5_
Organic carbon (Total)	~20.25 µg/mL	7.07 µg/mL
Elemental carbon (Total)	~19.85 µg/mL	2.27 µg/mL
Fe	~0.063 µg/mL	1.93 µg/mL
Zn	~0.052 µg/mL	0.24 µg/mL
Endotoxin	~0.5 EU/mL	0.41 EU/mL

## Data Availability

The data presented in this study are available on request from the corresponding author. The data are not publicly available due to the large size of raw data files.

## Supplementary Materials

The following supporting information can be downloaded at: https://www.mdpi.com/article/10.3390/jox15050142/s1. Supplementary Figure S1: Quantification of cell death by 7-AAD staining following fluorescent particle uptake assays in DEP- or PM_2.5_-exposed PMDC05 cells. Supplementary Table S1: Sampling conditions and chemical composition of PM_2.5_ samples collected using a large cyclone. References [47,48,49] are cited in the supplementary materials.
