# Supplementary material for: Sub-Toxic Exposure to DEPs and PM2.5 Impairs Dendritic Cell Function Through Intracellular Particle Accumulation
Source: J Xenobiot. 2025 Sep 8;15(5):142. doi: 10.3390/jox15050142 (PMC12452448; doi:10.3390/jox15050142)
Supplement: Supplementary file 1 [file jox-15-00142-s001.zip › jox-3791476-supplementary.pdf]

Supplementary Materials: Sub-Toxic Exposure to DEPs and $PM_{2.5}$ Impairs Dendritic Cell Function Through Intracellular Particle Accumulation

Yuki Nakahira [1], Daisuke Otomo [1], Tomoaki Okuda [2] and Akira Onodera [1,*]

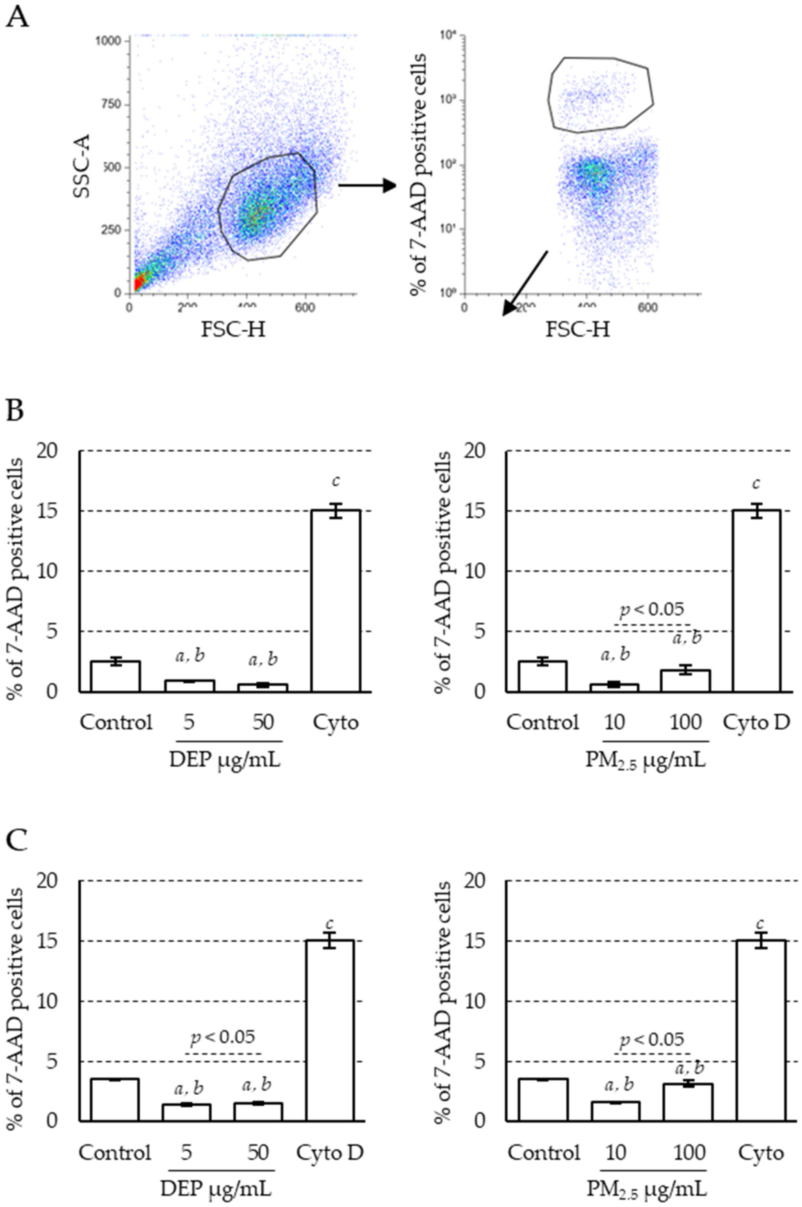

Figure S1. Quantification of cell death by 7-AAD staining following fluorescence particle uptake assays in DEP- or $PM_{2.5}$-exposed PMDC05 cells.

(A) Representative flow cytometry dot plots illustrating the dead cell gating strategy. Cells were initially gated based on SSC versus FSC, followed by the selection of 7-AAD–positive populations using FSC versus 7-AAD plots. The gated region indicates the dead cells. (B) Percentage of 7-AAD–positive cells after 24-hour exposure to 300-nm fluorescent silica particles. Cells were pretreated with PBS, DEP, $PM_{2.5}$ or 1 μM Cyto D. (C) Percentage of 7-AAD–positive cells after 24-hour exposure to 750-nm fluorescent polystyrene particles under the same pretreatment conditions as in (B). The Kruskal–Wallis test was used for statistical analysis,

followed by Steel's post hoc multiple comparison. Significant differences between groups: a $p < 0.05$ vs. control; b $p < 0.001$ vs. control; c $p < 0.001$ vs. Cyto D. Pretreatment with DEP or $PM_{2.5}$ resulted in significantly lower percentages of 7-AAD–positive cells than Cyto D, suggesting that particle-induced cytotoxicity may have occurred and resolved before the secondary particle challenge. Cyto D, a well-characterised actin polymerisation inhibitor [47], was used as a positive control for endocytosis inhibition. A concentration of 1 μM was selected based on preliminary experiments, which showed that 10 μM induced substantial morphological alterations (data not shown). These results confirm that the uptake assays were performed under conditions that minimised cytotoxic interference.

Table S1. The sampling conditions and chemical composition of $PM_{2.5}$ collected using a large cyclone.

This table summarises the sampling conditions and chemical composition of $PM_{2.5}$ collected using a large-volume cyclone (inner diameter ≈ 68 mm; HVS3, CS3 Inc., Sandpoint, ID), as reported by Alimov et al. (2022) [19]. Chemical analyses of the collected particles were performed using ion chromatography (IC), energy-dispersive X-ray fluorescence spectroscopy (EDXRF), induc-tively coupled plasma mass spectrometry (ICP MS), and carbon fractionation (EC/OC). The EDXRF method followed the proto-col described by Jing et al. (2022) [48], and EC/OC analysis was conducted according to Okuda (2013) [49]. Detailed analytical procedures are available in the referenced publications.

Sample Code: JP-$PM_{2.5}$-23corrected   Period: 2021/4/19~6/18

Sampling Time [min]	Total Air Volume [m3]	Particle Mass [mg]	PM conc [µg/m3]
86593	105015	1145,68	10,91

IC Ion (ppmw)							
Cl-	NO3-	SO42-	Na+	NH4+	K+	Mg2+	Ca2+
13899,9	42092,2	36769,7	11286,3	2295,8	5599,9	2483,8	16806,1

EDXRF (ppmw)	
Si	P
57712,6	6355,2

Bioaerosol
Endotoxin (EU/mg)
4,11

ICP-MS metal (ppmw)								
Mg	Al	S	K	Ca	Ti	V	Cr	Mn
4549,3	40016,2	15565,8	6601,0	18579,8	2023,2	52,9	200,7	543,2
Fe	Co	Ni	Cu	Zn	As	Se	Cd	Pb
19272,1	6,9	81,0	534,3	2345,3	17,7	13,7	2,8	200,7

Carbon (ppmw)					
OC1	OC2	OC3	OC4	OCP	OC (Total)
779,5	6142,4	43201,8	6530,8	14042,4	70696,9
EC1	EC2	EC3	EC (Total)		
6848,4	11335,1	4563,7	22747,2		
